# Supplementary figures and images for: The Target Therapy Hyperbole: “KRAS (p.G12C)”—The Simplification of a Complex Biological Problem
Source: Cancers (Basel). 2024 Jun 28;16(13):2389. doi: 10.3390/cancers16132389 (PMC11240669; doi:10.3390/cancers16132389)

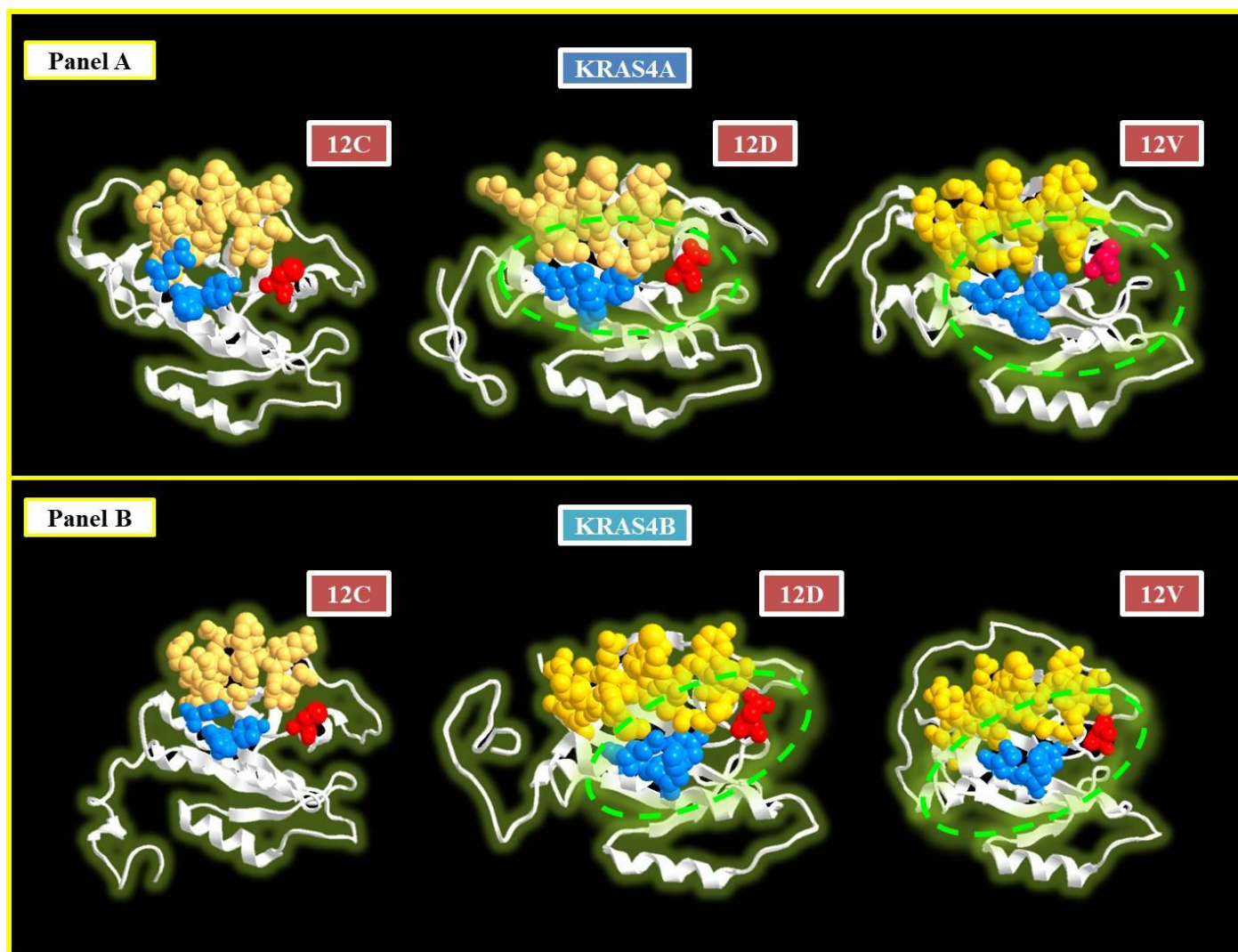

**Figure S1.** Pocket domains p-G12V and p.G12D.

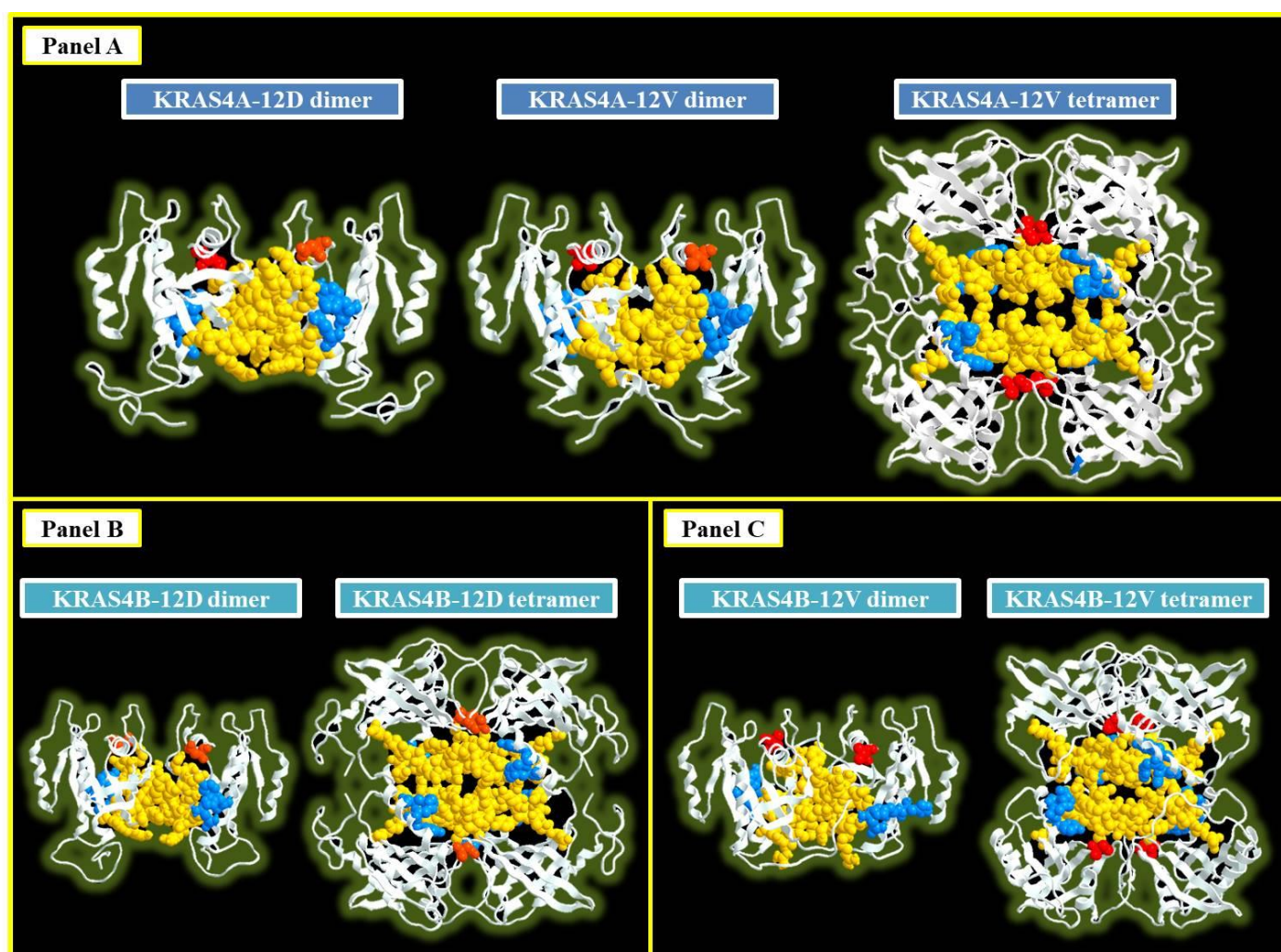

**Figure S2.** Aggregation propensity analysis for p-G12V and p.G12D.

Supplement: Supplementary file 1 [file cancers-16-02389-s001.zip › cancers-3052139-supplementary.pdf]
